# Conventional Rehabilitation Therapy Versus Telerehabilitation in Cardiac Patients: A Comparison of Motivation, Psychological Distress, and Quality of Life

**DOI:** 10.3390/ijerph16030512

**Published:** 2019-02-12

**Authors:** Helle Spindler, Kasper Leerskov, Katrine Joensson, Gitte Nielsen, Jan Jesper Andreasen, Birthe Dinesen

**Affiliations:** 1 Department of Psychology and Behavioral Sciences, Aarhus University, 8000 Aarhus, Denmark; hellesp@psy.au.dk; 2 SMI®, Department of Health Science and Technology, Aalborg University, 9200 Aalborg East, Denmark; kkl@hst.aau.dk; 3 Department of Micro- and Nanotechnology, The Technical University of Denmark, 2800 Kgs. Lyngby, Denmark; katrj@nanotech.dtu.dk; 4 Department of Cardiology, Vendsyssel Hospital, 9800 Hjoerring, Denmark; gn@rn.dk; 5 Department of Cardiothoracic Surgery, Aalborg University Hospital, 9000 Aalborg, Denmark; jja@rn.dk; 6 Department of Clinical Medicine, Aalborg University, 9000 Aalborg, Denmark; 7 Laboratory of Welfare Technologies - Telehealth and Telerehabilitation, Department of Health Science and Technology, Aalborg University, 9200 Aalborg East, Denmark

**Keywords:** telerehabilitation, cardiac rehabilitation, anxiety, depression, psychological distress, motivation

## Abstract

Telerehabilitation (TR) has gained attention as a promising rehabilitation format. Our study examined how patients responded to TR and whether it provided adequate support for their lifestyle changes and self-care efforts when compared to conventional rehabilitation (CR). Cardiac patients (*n* = 136) were randomly assigned to a TR or CR group. The TR group was provided with relevant health care technology for a period of three months, and both groups filled in questionnaires on their motivation for lifestyle changes and self-care psychological distress, and quality of life at 0, 3, 6, and 12 months. Patients in both groups were found to be equally motivated for lifestyle changes and self-care (*p* < 0.05) and they experienced similar levels of psychological distress and quality of life. TR is comparable to conventional rehabilitation in motivating patients, preventing psychological distress and improving quality of life. Although we observed an initial increase in autonomous motivation in the telerehabilitation group, this positive difference in motivation does not last over time. As such, neither rehabilitation format seems able to ensure long-term motivation. Therefore, TR may serve as a viable replacement for conventional rehabilitation when considered relevant. Further research is needed to enhance long-term motivation, and maybe telerehabilitation can help to achieve this.

## 1. Introduction

Cardiovascular disease (CVD) is the leading cause of death across Europe today [1]. Developing rehabilitation programs to address lifestyle changes and improve self-care in CVD is a key factor in reducing mortality rates, as effective rehabilitation has been associated with a reduced symptom burden, improved quality of life, reduced psychological distress (anxiety and depression), and decreased mortality [2,3,4]. As such, rehabilitation is a key factor in improving the overall course of CVD. However, despite the benefits associated with rehabilitation, a large group of patients do not participate in the rehabilitation programs offered to them, some due to sociodemographic factors, whereas others refuse to participate because of difficulties travelling long distance or having to work when rehabilitation is offered [5,6,7,8,9]. As a result, a large number of patients do not benefit from the possible advantages of rehabilitation [3,7].

Telerehabilitation therapy may be considered as part of telehealth, a globally increasing method of delivering health care services using information and communication technologies [10]. While there are many studies of telerehabilitation, there is a lack of long-term studies of the effect of telehealth, as recent reviews suggest a possible publication bias and lack of long-term follow-up, which is especially pertinent to assessing rehabilitation programs [10,11]. As such, Telerehabilitation (TR) is defined as the delivery of rehabilitation services via information and communication technologies [12]. TR is intended to overcome some of the obstacles of traditional rehabilitation, as digital technology (a) may be provided in the patient’s own home, (b) covers long distances, and (c) offers unlimited temporal flexibility [8]. As such, TR may prove to be a better fit with the patient’s lifestyle and thereby increase the patient’s self-management [13]. Although TR is a relatively new format, studies on home-based TR have been promising in terms of achieving levels of improvement comparable to conventional rehabilitation (CR) on clinical measures and quality of life (QoL), anxiety, and depression [14,15], as well as improving overall adherence to rehabilitation [6]. These results seem to refute the concern that patients participating in TR may fare worse in terms of psychological distress and quality of life because they have less personal contact with health personnel compared to patients in CR [8,16]. In addition, studies have shown that technology such as pedometers may increase motivation for walking [17], while interactive web resources may be used for patient education and developing e-health literacy skills. Taken together, these studies suggest that TR may be a promising option for future health systems, particularly as it increases the availability of individually tailored solutions, thus supporting the new health care paradigm of personalized medicine [10].

Individualized rehabilitation interventions may increase patients’ perceived controllability of the disease and thereby perceived self-efficacy in terms of self-care [18]. The patient’s sense of control may be reinforced if the patient is invited to participate in setting realistic goals for outcomes in rehabilitation [19]. This suggests that cooperating with the patient in setting goals for rehabilitation and self-care may in turn increase adherence to these goals over time [19], as well as increase QoL [20]. Failure to adhere to lifestyle changes and desired goals when the rehabilitation program ends is a well-known problem in cardiac rehabilitation [21]. Designing more individualized rehabilitation programs may be one strategy to address this issue and be considered alongside the incorporation of technology [10].

Recently, it has been stressed that designing rehabilitation programs supported by new technology for behavioral change should be informed by theory [22], as this may increase the effectiveness of behavior change interventions. Self-Determination Theory (SDT) [23] focuses on individual motivation and the role of fulfilling basic needs such as autonomy, competency, and relatedness in building the kind of intrinsic motivation needed to self-regulate and sustain health behavior independently over time. According to SDT, a person who considers their health behavior to be in accordance with their own values and beliefs (autonomy), feels that they possess the knowledge and skills to perform this health behavior (i.e., competency, a term closely related to self-efficacy) and who feels supported by others (relatedness) will achieve the kind of intrinsic motivation that will enable them to maintain their health behavior over time [23]. Individualization of rehabilitation programs by actively involving the patient in setting goals may thus directly increase the patient’s experience of autonomy and in turn their motivation, as suggested by Jansson et al. [19]. However, as SDT stipulates, successful motivation requires a sufficient fulfillment of all three needs simultaneously. A recent study examining SDT parameters in patients undergoing cardiac rehabilitation showed an association between SDT motivational factors and improvements in patients’ physical and psychological health [24]. Despite these promising results, using SDT as a framework for motivating patients to engage in appropriate health behavior may not be sufficient to ensure lasting lifestyle changes and enhanced QoL. In addition, long-term motivation for self-care may diminish due to anxiety and depression, which has been associated with an impaired QoL [25,26,27]. Finally, psychological distress and impaired QoL may negatively impact self-care [28], as well as motivation [29]. These findings suggest the importance of addressing both motivation and possible psychological distress in order to support patients’ efforts at improving health behavior.

Focusing on these diverse challenges, the Teledialog project developed a personalized cardiac TR program tailored to each individual patient according to their specific rehabilitation needs and goals. The individualized rehabilitation program included counting of steps, measurement of vital values such as blood pressure, pulse and weight, access to an interactive web portal and additional rehabilitation measures designed to supports the patients’ motivational needs. In addition to using SDT as a theoretical background, we used user-driven innovation to ensure the feasibility of our intervention design, as user-driven innovation increases the probability of patients adopting the technology [30]. 

In the current study, we evaluated this TR program in terms of its ability to support patients’ motivational needs and in terms of how patients in the telerehabilitation program fared psychologically. The aim of our study was to one, examine whether telerehabilitation was superior to CR with regards to motivating participants and secondly, to determine the psychological distress level of cardiac patients undergoing the proposed TR program compared to patients in a CR regime.

## 2. Materials and Methods 

### 2.1. Ethical Approval

This study is a psychological sub study within the larger, Teledialog project, which examined various outcomes of TR using a randomized controlled trial design. In the present paper, we present our specific sub study; further details of the overall research project may be found at clinicaltrials.gov (ClinicalTrials.gov Identifier: NCT01752192) [31]. All subjects gave their informed consent for inclusion before they participated in the study. The study was conducted in accordance with the Declaration of Helsinki, and the protocol approved by the Ethics Committee of North Jutland (N-20120051).

### 2.2. Participants

**Figure 1:**
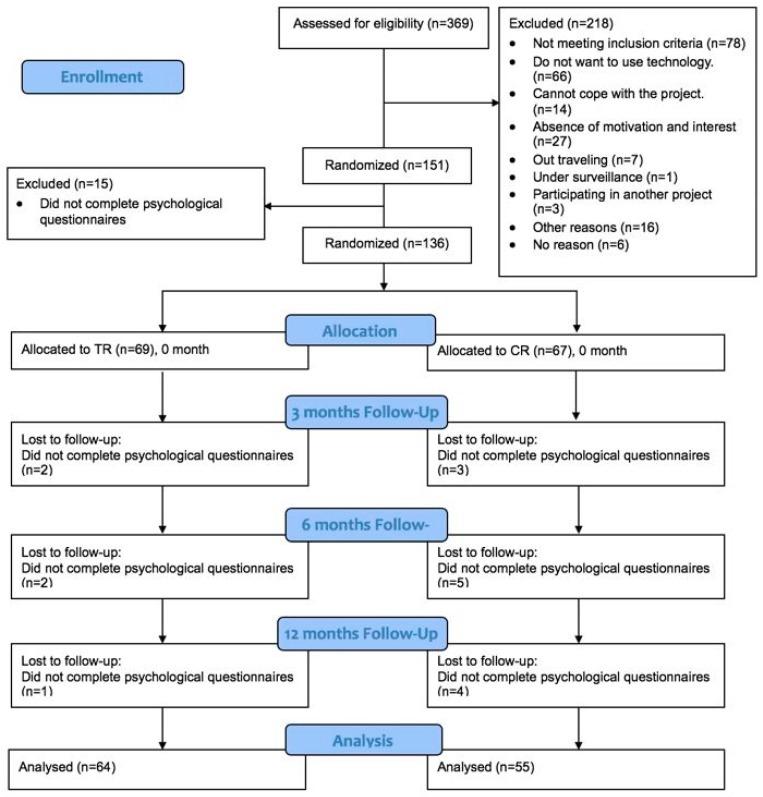
Consort diagram in the Teledialog telerehabilitation program.

The recruitment of participants was carried out by a project nurse following the patients’ discharge from the healthcare centers or hospitals. The inclusion criteria for the overall study were: 18 years of age or above, a diagnosis of coronary artery bypass, valve surgery, heart failure or artery sclerosis, ability to understand Danish, and ability to use digital technology (including using a mobile network). Out of 369 participants assessed for eligibility, 151 participants were enrolled in the Teledialog project, please see Figure 1. Of these, 136 fulfilled to all psychological measures and were assessed as eligible for inclusion in this sub-study. Patients who declined participation in the psychological sub-study were more likely to be female, have a higher level of education, and be diagnosed with heart valve disease, and less likely to be diagnosed with acute coronary syndrome (results not shown).

After inclusion, all participants were randomized into either the telerehabilitation (TR) group or the conventional rehabilitation (CR) group using an automatically generated list with an equal number in each group so as to ensure a randomized, but even distribution.

### 2.3. Procedure

All included patients filled in a questionnaire at baseline on enrollment, and at 3, 6, and 12 months. The baseline measures were taken prior to engaging in the TR program, in which they were given several digital devices; the 12 weeks follow-up was filled in just after returning all the devices except the Fitbit, and the remaining two questionnaire packets were completed at 6 and 12 months, respectively. Non-responders were requested to fill in their questionnaires using a maximum of two reminders.

### 2.4. The Intervention

In both groups, patients and their partners/spouses were offered equal access to non-formalized personalized feedback by health care staff as part of the rehabilitation program. As such, the groups did not differ in the formal provision of contact, support, and feedback offered by health care staff. Although differences occurred between individual patients, these differences were not a result of group allocation. Rather, they reflected the patients’ individual needs and characteristics.

#### 2.4.1. TR Group

**Figure 2:**
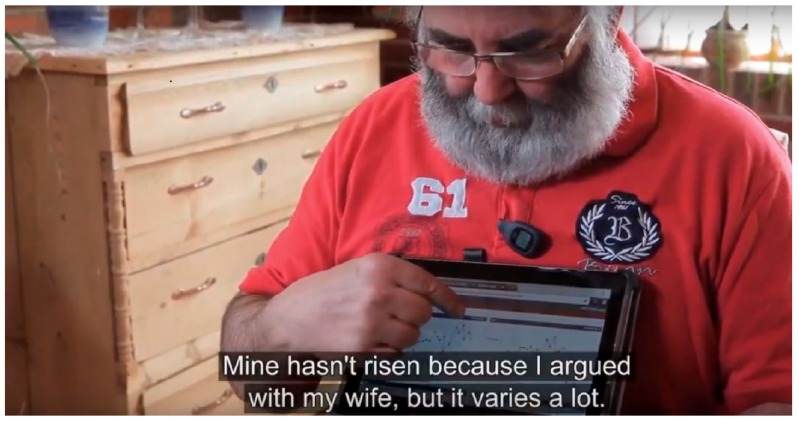
YouTube Video about Teledialog also illustrating the interactive web portal.

After enrollment, an individualized TR program lasting 12 weeks was designed for each participant in the TR group. Each participant was given a Teledialog toolbox containing technology for the Teledialog project (a blood pressure monitor, scales, heart rate monitor, and a digital step counter, as well as a tablet PC with a mobile network). They were instructed in the use of the technology in the Teledialog toolbox, such as measuring blood pressure, pulse, weight, and steps, uploading data, as well as using a digital rehabilitation plan (e-rehabilitation plan). Accessing their personal health record and measurements through the e-rehabilitation plan, the participating patients were able to monitor their personal data at all times using the tablet PC. In addition, the e-rehabilitation plan enabled communication and sharing of information between healthcare staff, patients, and partners. Furthermore, the interactive web portal ActiveHeart, containing educational materials centered on living with CVD and using written text and video, was used as the access point for the e-rehabilitation plan. In this way, patients and their partners had a single access point for all health information and communication regarding the patient. Both patient and partner could access this platform, with the partner having their own login. Figure 2 is a screendump from the YouTube video about the Teledialog project [32].

To ensure that patients and their partners were able to use the Teledialog technology appropriately, a monitoring visit was carried out by a research assistant after the second week of the program. During the entire 12-week intervention period the healthcare staff monitored the measured values twice a week and had contact with the individual participant if needed in order to discuss the progress of their rehabilitation activities or any abnormal measures. Each patient received guidance in physical training, education in lifestyle changes such as dietary guidance, psychoeducation, and smoking cessation.

#### 2.4.2. The Control Group

The control or CR group was enrolled in a non-technology-based conventional cardiac rehabilitation program necessitating visits to the healthcare center or hospital. This program resembled the rehabilitation usually offered according to the European Guidelines on cardiac rehabilitation [33]. Like the TR patients, each patient in the control group also followed a 12-week program consisting of physical training, education in lifestyle changes such as dietary guidance, psychoeducation, and smoking cessation. The rehabilitation program consisted of both individual guidance and group sessions. Blood pressure, pulse, and weight were measured at the time of enrollment in the program and again 12 weeks later, at the end of the rehabilitation program.

### 2.5. Measures

Sociodemographic data (age and gender) and clinical data (acute coronary syndrome, bypass surgery, health valve disease, heart failure, hypertension, diabetes, obesity) were collected through self-reporting or from each patient’s medical journal.

#### Psychological Measures

*Health-Care: Self-Determination Theory Packet*

Autonomy, competency, and relatedness were measured using the Health-Care, Self-Determination Theory Packet (HC-SDT). The SDT Packet consists of three questionnaires: 1) the Treatment Self-Regulation Questionnaire (TSRQ), which measures autonomy (15 items); 2) the Perceived Competence Scale (PCS), which measures competency (4 items); and 3) the Health Care Climate Questionnaire (HCCQ), which measures relatedness (15 items). All items are statements rated on a 7-point Likert scale (from ‘Not at all true’ to ‘Very true)’. The TSRQ is divided into three independent subscales: autonomous regulatory style (average of six items), controlled regulatory style (average of 6 items) and amotivation (average of three items). Scores for the PCS and the HCCQ are an average of all items. However, the HCCQ may be used in its full version (15 items) or in a shorter version (six items). In our study, we used the shorter version. All statements were rephrased in order to capture the participant’s overall experience of autonomy, competency, and relatedness in relation to the rehabilitation process [34].

*Hospital Anxiety and Depression Scale*

The Hospital Anxiety and Depression Scale (HADS) is a 14-item self-reporting measure, consisting of two 7-item subscales measuring anxiety and depressive symptoms devoid of somatic symptoms [35]. All responses are indicated on a four-point Likert scale from 0–3 (score range 0–21). The two subscales have been shown to be internally consistent, as measured by Cronbach’s α: HADS-A = 0.80; HADS-D = 0.81 [36]. Moreover, a review of 15 studies showed HADS to be a valid and reliable instrument with Cronbach’s α for HADS-A ranging from 0.68–0.93 and for HADS-D ranging from 0.67–0.90 [36]. This review also showed ≥8 on both subscales to be an optimal cut-off point as indication of likely psychopathology, with sensitivity and specificity ranging between 0.70 and 0.90 for most reviewed studies [37]. Despite controversies in the literature regarding the diagnostic validity of the two subscales of the HADS, we used the original subscales, as we wanted to compare levels of psychological distress across our two intervention arms. Hence, we used the HADS as a general indicator of psychological distress rather than as a diagnostic tool [38,39,40].

*Short-Form Health Survey (SF-36)*

The SF-36 is a generic measure of health-related quality of life (HRQL) that enables comparisons of HRQL across different somatic diseases as well as with healthy populations [37]. The SF-36 consists of 36 items grouped into eight subscales: physical functioning, role physical functioning, role emotional functioning, mental health, vitality, social functioning, bodily pain, and general health. Each subscale is measured on a scale from 0–100 and is a purely descriptive measure without a preference-based evaluation of the health state. A high score indicates good HRQL, with the exception of the bodily pain subscale, whereas a high score represents the absence of pain. The SF-36 has proven to be a valid and reliable instrument, with Cronbach’s α for the 8 subscales ranging from 0.78 (general health) to 0.93 (physical functioning) [41].

*Statistical Procedure*

Prior to analysis, data quality was examined, and missing data was handled by case-wise exclusion, resulting in a final sample of 136 patients. Cases were excluded from the psychological sub-study if data was insufficient to allow calculation of summed scores on psychological measures.

Differences between the two rehabilitation groups on sociodemographic and clinical variables at baseline were examined using Fisher’s exact, *t*-test or *chi*^2^, as appropriate. To examine differences between groups over time, we conducted a series of 2 (group) × 4 (time) ANOVAs using as dependent variables the subscales of the Health-Care, Self-determination Theory Packet, the HADS and the SF-36. In addition, we calculated Cohen’s d_Cohen_ in order to estimate the magnitude of change between groups and over time in each group. All analyses were carried out using SPSS version 24.0.0.0 (IBM, New York, NY, USA).

## 3. Results

### 3.1. Baseline Characteristics

Table 1 displays the baseline sociodemographic, clinical, and psychological variables for the TR and CR groups used in this study. In designing the study and in accordance with previous findings in this sample [42], we found no differences in sociodemographic or clinical variables across groups. At baseline, there were no significant differences between the two groups on any of our measures of psychological distress, although a trend could be argued for a difference in depression and anxiety, as the study was underpowered to detect such differences (p_anxiety_ = 0.07, d_Cohen(anxiety)_ = 0.30, β_anxiety_ = 0.40; p_depression_ = 0.07, d_Cohen(depression)_ = 0.32, β_depression_ = 0.44) (see Table 1). A significant baseline difference was also found for autonomous motivation with our CR group reporting more autonomous motivation compared to our TR group (*p* = 0.049), as well as a similar trend for controlled motivation (p_controlled motivation_ = 0.08, d_Cohen(controlled motivation)_ = 0.31, β_controlled motivation_ = 0.42) (Table 1).

We also conducted an analysis of participant dropout over the course of the study. We found that patients who dropped out were more likely to suffer from heart failure than those who completed the study (results not shown). This is not surprising, considering the impact heart failure may have on a person’s physical and mental well-being. However, we chose not to include these findings in a diagnostic subgroup analysis of our overall aims, as the number of patients in each group was too small for robust analysis. 

### 3.2. Motivation

**Figure 3:**
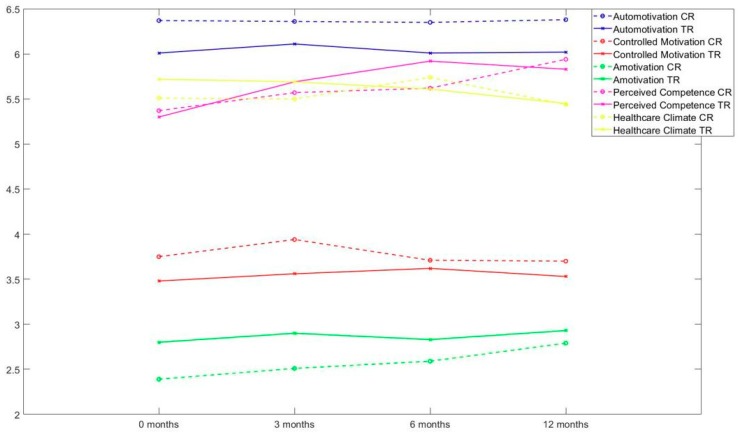
Motivational changes in the TR and CR groups over time.

We used ANOVA with repeated measures of motivational factors in order to examine our first aim. We found an initial difference in autonomous motivation between the two groups, with the CR group showing higher levels of autonomous motivation over time (see Table 2 and Figure 3.). However, despite an initial increase in autonomous motivation in the TR group, the overall levels of autonomous motivation remained relatively stable over time across the two groups (F_2.78_ = 0.21, *p* = 0.88). In addition, both groups significantly improved their levels of perceived competence over time (F_2.85_ = 8.68, *p* < 0.01), but at comparable levels, indicating that both groups benefitted equally in terms of increased competence, irrespective of group allocation. No other analyses of the effects or interactions of motivational factors showed significant results (see Table 2).

### 3.3. Anxiety, Depression, and Quality of Life (QoL)

To examine whether TR was associated with more psychological distress and impaired QoL, we conducted ANOVA with repeated measures of anxiety and depression. The ANOVA revealed that the level of anxiety (F_2.69_ = 10.70, *p* < 0.01) and depressive (F_2.49_ = 3.40, *p* = 0.03) symptoms decreased at comparable levels in both groups over time. Analyses of QoL scores indicate the same pattern of change, with physical, social, role physical, role emotional, mental health, vitality and pain improving comparably over time (all *p*’s < 0.01), whereas no significant changes were evident for the general health subscale (see Table 2).

Overall, our results show no significant differences between the two rehabilitation groups with regard to the level of anxiety and depressive symptoms as well as quality of life experienced by patients. This finding is supported by examining the effect sizes within each group, which indicated that anxiety and depressive symptoms showed comparable levels of remission in both groups over time, with effect sizes (ES) of a small to medium size (ES: d_Cohen(TR)(anxiety/depression)_ = 0.47/0.24; d_Cohen(CR)(anxiety/depression)_ = 0.30/0.27). A similar result was found for all subscales on the SF-36 (ES: d_Cohen(TR)_ = −0.49−(−0.63); d_Cohen(CR)_ = −0.31−(−0.55)), with the exception of the general health subscale (d_Cohen(TR/CR)_ = 0.00/−0.01), as also indicated by test results in Table 2.

## 4. Discussion

Our results indicate that TR is comparable to CR with regards to motivation, psychological distress, and QoL. Although there were differences in autonomous motivation across groups, these were a priori and cannot be attributed to any difference in the two rehabilitation formats. Initially, the TR group may have been less motivated because they had been randomized to the TR condition instead of the face-to-face CR. To our knowledge, this is the first study to show that psychological distress decreases at comparable levels in both TR and CR. Our findings suggest that TR may not be associated with a greater risk of psychological distress compared to conventional, on-site rehabilitation.

In this study, we applied user-driven innovation to ensure the feasibility of our intervention design, as user-driven innovation increases the probability of patients adopting the technology as a useful tool [30]. This motivational strategy in user-driven innovation is in accordance with how SDT conceptualizes motivation, especially in terms of autonomy and competency. Hence, in the user-driven design process, users may call attention to design issues based on their values (autonomy) and express their needs in terms of features that may increase their competency with regards to disease management. We thereby aimed to optimize the ability of our TR program to increase autonomous motivation. However, we achieved this goal only initially (see Figure 3) in our TR group, suggesting that although our TR program initially increased autonomous motivation, this increase was dependent on the availability of the technology, not on patients having internalized motivation for rehabilitation goals. In other words, the increase in autonomous motivation was more likely associated with external motivation derived from the technology. This suggests that in order for patients to internalize autonomous motivation so that they remain motivated for continued self-care and lifestyle changes, a more extensive intervention is needed that more effectively addresses the patient’s ideas and values.

In future rehabilitation programs, it may be worthwhile to consider interventions such as the Gothenburg Patient Centered Care Program (GPCC), which focuses explicitly on engaging patients in decision-making regarding their rehabilitation goals through individualization [18,19]. Another method might be motivational interviewing, the aim of which would be to increase autonomy and stimulate overall motivation [43]. Engaging patients in decision-making to obtain individualization constitutes a more passive approach, in that it is not aimed at changing the patient’s values directly, whereas motivational interviewing aims to specifically address, and hopefully change, the patient’s values. Motivational interviewing may thus be said to actively address autonomy as conceptualized within the theoretical framework of SDT [43]. However, as suggested by Paquet et al. [44], rehabilitation programs in general may overlook an important barrier preventing patients from internalizing autonomous motivation: namely, that the patient’s immediate needs may be centered on stress management rather than lifestyle and behavioral change. If patients experience high levels of stress, their readiness to change and their continued autonomous motivation may be diminished. As such, motivational interviewing, which focuses on assisting the patient in addressing their possible conflicting motivation, including experienced stress, may be superior in supporting the patient’s decisional process [43], although this focus could also be adopted when working within the GPCC program.

Despite the rehabilitation format having no significant impact on motivation, both our rehabilitation groups increased their competency significantly over time. Apparently, the rehabilitation format does not influence patients’ ability to increase their knowledge and skills regarding self-care and lifestyle changes. These findings also accord with other findings from the Teledialog project showing that the interactive web resource increased e-health literacy [45]. As competency is a key factor in self-care, this result is indeed positive, as it suggests that the immediate task of the rehabilitation program is also successful when using digital technology. However, it also suggests that both CR and TR programs are well-suited to increase patients’ competency levels. On the other hand, neither of these rehabilitation formats were as effective in enhancing the other factors that increase motivation, such as autonomy and relatedness. 

TR is often understood as involving little or no personal contact with health personnel [8]. This stereotypical understanding results in a common misconception that TR is inferior in addressing or identifying psychological distress in patients, as psychological distress is often identified through personal contact. The implication of this presumed shortcoming is that TR could be associated with a greater risk for psychological distress. Our findings suggest that this is not the case. Our two groups showed comparable levels of psychological distress over time. Hence, in this first study, directly comparing psychological distress across rehabilitation formats, the level of psychological distress is not associated with the rehabilitation format. These findings are not necessarily surprising, as personal contact with health personnel often fails to address psychological distress issues [46]. As such, our results are in line with the findings by Cartwright et al. [14] from Whole System Demonstrator, who concluded that telehealth is neither superior nor inferior to face-to-face contact with regards to QoL or psychological distress. However, the issue may be of a different order, as underlined by Paquet et al.’s [44] findings. Pacquet et al. found that patients were initially preoccupied with managing their stress rather than engaging in rehabilitation activities. Stress management was simply a higher priority item for these patients. When psychological resources are spent trying to handle stress, there is no surplus energy or motivation to engage in lifestyle changes and other rehabilitation activities. Taken together, these findings suggest that future TR programs should incorporate some form of screening for psychological distress that could improve the program’s ability to identify and subsequently offer appropriate psychological interventions to alleviate psychological distress in accordance with current recommendations. Addressing patients’ psychological distress may free up necessary psychological resources that could enable the patient to mobilize the motivation resources and engage fully in rehabilitation activities.

In line with Cartwright et al. [14], we also find comparable levels of QoL over time in both groups, with QoL increasing at clinically relevant levels in both groups. The implication here is that both types of rehabilitation formats support patients’ efforts to improve their QoL. The type of rehabilitation format seems not to be associated with any potential improvement in QoL achieved through rehabilitation. Thus, TR may be the superior choice when trying to engage those patients who live far from the hospital or health centers, those who must attend to work tasks, or patients otherwise unable or unwilling to attend face-to-face rehabilitation on a regular basis. As rehabilitation attendance is a significant problem in cardiac populations, alternative flexible rehabilitation formats such as TR, in so far as they provide comparable results, may help increase the number of patients engaging in and completing rehabilitation programs.

Taken together, our findings suggest that successful TR programs must be able to motivate the patient for the long haul. They need to build up the kind of intrinsic motivation for long term self-care and necessary lifestyle changes [21]. In addition, although rehabilitation programs rarely focus on psychological distress, the reduced personal contact with health care personnel must not result in psychological distress being overlooked. Nor should patients become psychologically distressed because they lack the opportunity to discuss their worries with health care staff. In sum, TR solves problems of distance, flexibility, working life and may make certain rehabilitation routines more convenient. However, TR can never be used as a replacement for the necessary contact between the patient and health care providers.

### Limitations

Although our results are promising in terms of using TR as a feasible alternative to face-to-face rehabilitation, our results should be interpreted with the following limitations in mind. First of all, our sample size may have been too small to identify relevant differences in psychological distress, QoL and motivation, as our TR group generally fared worse on almost all psychological variables compared to our CR groups. However, the effect sizes for each group indicate that the magnitude of change was comparable across the two groups and these differences in baseline characteristics may be due to the fact that patients were informed of their allocation prior to completing the psychological assessment survey. Having been allocated to a less familiar rehabilitation format may have had a negative psychological impact, which can be identified in these measures. Secondly, the generalizability of our results may be compromised by the fact that there were significant differences in those who participated in our study and those who did not. Thirdly, due to our sample size and dropouts, we did not examine individual characteristics by focusing on whether some patients were more likely than others to benefit from telerehabilitation. Lastly, our results are based on a small sample, and further research is needed to test whether our results can be replicated on a larger scale.

## 5. Conclusions

Taken together, our results suggest that there are no differences between conventional rehabilitation and telerehabilitation in terms of motivating patients to engage in rehabilitation activities. Although we observed an initial increase in autonomous motivation in the telerehabilitation group, this positive difference in motivation does not last over time. As such, neither rehabilitation format seems able to ensure long-term motivation. Telerehabilitation is not a ‘magic bullet’. It works as well (or as poorly, in terms of motivation) as does conventional rehabilitation therapy. Contrary to received assumptions, our results indicate that telerehabilitation is not associated with increased psychological distress. Psychological distress decreases at comparable levels regardless of whether it takes place under conventional rehabilitation or telerehabilitation. Finally, patients in both groups experience QoL at comparably similar levels. In sum, our findings indicate that telerehabilitation may be a viable option for patients who might otherwise either decline to participate or only partially participate rehabilitation because of time restraints or distance. Further research is needed to enhance long-term motivation and perhaps there is a way of tweaking telerehabilitation to achieve this. 

## Figures and Tables

**Table 1:** Statistical comparison of baseline characteristics between groups.

Variable	Tele-rehabilitation (TR) *n* = 69	Conventional Rehabilitation (CR) *n* = 65	Total Sample	*p*-Value
**Sociodemographic variables**				
Age mean (SD)	61.86 ± 12.46	62.68 ± 11.95	62.25 ± 12.17	0.70
Males n (%)	54 (50.5)	53 (49.5)	107 (79.9)	0.64
Education	46 (50)	46 (50)	92 (71.3)	0.49
Work	28 (53.8)	24 (46.2)	52 (44.1)	0.93
**Clinical variables**				
Acute Coronary Syndrome (ACS) n (%)	42 (50.6)	41 (49.4)	83 (61.9)	0.80
Bypass n (%)	12 (57.1)	9 (42.9)	21 (15.7)	0.57
Heart Valve n (%)	12 (66.7)	6 (33.3)	18 (13.4)	0.17
Heart Failure n (%)	18 (48.6)	19 (51.4)	37 (27.6)	0.68
Hypertension n (%)	4 (66.7)	2 (33.3)	6 (4.5)	0.45
Diabetes (type 1+2) n (%)	3 (75.0)	1 (25.0)	4 (3.0)	0.34
Obesity n (%)	3 (60.0)	2 (40.0)	5 (3.7)	0.70
**Motivation**				
Autonomous Motivation	6.00 ± 0.97	6.31 ± 0.84	6.15 ± 0.92	0.049
Controlled Motivation	3.51 ± 1.51	3.96 ± 1.43	3.73 ± 1.50	0.08
Amotivation	2.77 ± 1.54	2.61 ± 1.53	2.67 ± 1.53	0.55
Perceived Competence	5.22 ± 1.18	5.39 ± 1.24	5.30 ± 1.21	0.41
Healthcare Climate	5.69 ± 1.15	5.50 ± 1.43	5.59 ± 1.29	0.39
**Psychological variables**				
Anxiety	7.10 ± 4.48	5.82 ±3.41	6.54 ± 4.04	0.07
Depression	5.58 ± 4.05	4.46 ± 2.95	5.07 ± 3.57	0.07
Physical functioning	58.99 ± 28.14	65.54 ± 26.39	62.10 ± 27.40	0.17
Social functioning	73.01 ± 28.57	79.04 ± 23.71	75.74 ± 26.25	0.19
Role physical functioning	35.87 ± 41.89	41.15 ± 42.26	38.05 ± 41.83	0.47
Role emotional functioning	56.52 ± 42.90	57.95 ± 44.60	57.11 ± 43.30	0.85
Mental Health	66.72 ± 22.07	71.82 ± 19.47	69.09 ± 20.81	0.16
Vitality	46.16 ± 27.64	52.15 ± 24.62	48.86 ± 26.18	0.19
Pain	58.72 ± 27.55	61.14 ± 28.46	59.62 ± 27.83	0.62
General Health	59.42 ± 23.56	63.60 ± 21.87	61.18 ± 22.76	0.29

All categorical data were analyzed using *chi*^2^, whereas continuous data was analyzed using *t*-tests. Numbers may not add up to 100, due to missing values.

**Table 2:** Analysis of changes over time in motivation, anxiety, depression, and quality of life for Telerehabilitation (TR) and Conventional Rehabilitation (CR) groups.

Time Point	0 mos. Mean (S.D.)	3 mos. Mean (S.D.)	6 mos. Mean (S.D.)	12 mos. Mean (S.D.)	F Time Group Interaction	*p*-Value
**Motivation**						
Autonomous Motivation TR _(*n* = 64)_ CR _(*n* = 55)_	6.01 (0.98) 6.37 (0.85)	6.11 (0.91) 6.36 (0.86)	6.01 (1.16) 6.35 (.76)	6.02 (1.01) 6.38 (0.77)	F_2.78_ = 0.21 F = 5.09 F_2.78_ = 0.09	0.88 0.03 0.85
Controlled Motivation TR _(*n* = 64)_ CR _(*n* = 55)_	3.48 (1.53) 3.75 (1.47)	3.56 (1.41) 3.94 (1.37)	3.62 (1.55) 3.71 (1.42)	3.53 (1.54) 3.70 (1.73)	F_2.86_ = 0.55 F = 0.64 F_2.86_ = 0.54	0.64 0.33 0.65
Amotivation TR _(*n* = 64)_ CR _(*n* = 55)_	2.80 (1.56) 2.39 (1.47)	2.90 (1.47) 2.51 (1.23)	2.83 (1.62) 2.59 (1.37)	2.93 (1.71) 2.79 (1.54)	F_3_ = 1.28 F = 1.73 F_3_ = 0.45	0.28 0.19 0.72
Perceived Competence TR _(*n* = 64)_ CR _(*n* = 55)_	5.30 (1.16) 5.37 (1.29)	5.69 (1.27) 5.57 (1.27)	5.92 (1.11) 5.62 (1.23)	5.83 (1.23) 5.94 (1.14)	F_2.85_ = 8.68 F = 0.11 F_2.85_ = 1.31	0.00 0.74 0.27
Healthcare Climate TR _(*n* = 64)_ CR _(*n* = 55)_	5.72 (1.18) 5.51 (1.52)	5.69 (1.28) 5.50 (1.47)	5.61 (1.42) 5.74 (1.32)	5.45 (1.51) 5.44 (1.57)	F_2.71_ = 1.24 F = 0.11 F_2.71_ = 0.81	0.30 0.74 0.48
**Psychological**						
Anxiety TR _(*n* = 64)_ CR _(*n* = 55)_	7.06 (4.49) 5.93 (3.67)	5.42 (4.33) 5.15 (3.90)	5.38 (4.18) 5.11 (4.02)	5.06 (3.92) 4.78 (3.85)	F_2.69_ = 10.70 F = 0.56 F_2.69_ = 1.04	0.00 0.45 0.37
Depression TR _(*n* = 64)_ CR _(*n* = 55)_	5.53 (4.01) 4.51 (3.06)	4.38 (4.16) 4.20 (3.69)	4.44 (4.52) 4.16 (3.53)	4.47 (4.78) 3.64 (3.25)	F_2.49_ = 3.40 F = 0.89 F_2.49_ = 0.83	0.03 0.35 0.46
**Quality of Life**						
Physical functioning TR_(*n* = 64)_ CR_(*n* = 55)_	60.63 (28.32) 67.09 (25.25)	73.59 (26.19) 75.82 (21.73)	74.61 (25.33) 75.45 (24.21)	74.38 (26.13) 76.36 (23.44)	F_1.83_ = 18.83 F = 0.50 F_1.83_ = 0.91	0.00 0.48 0.40
Social functioning TR _(*n* = 64)_ CR _(*n* = 55)_	71.68 (28.97) 78.18 (24.44)	78.71 (25.26) 86.82 (16.57)	83.20 (22.63) 85.45 (21.08)	85.35 (21.31) 85.45 (21.21)	F_2.63_ = 9.49 F = 1.65 F_2.63_ = 1.45	0.00 0.20 0.23
Rolephysical functioning TR _(*n* = 64)_ CR _(*n* = 55)_	37.11 (42.95) 40.91 (43.93)	50.78 (41.54) 55.00 (42.60)	53.52 (44.96) 54.55 (45.41)	61.33 (42.71) 65.45 (63.01)	F_2.77_ = 10.23 F = 0.26 F_2.77_ = 0.06	0.00 0.61 0.98
Roleemotional functioning TR _(*n* = 64)_ CR _(*n* = 55)_	57.29 (42.61) 56.97 (44.75)	65.10 (42.59) 70.30 (38.85)	67.19 (40.93) 72.12 (41.46)	76.56 (34.47) 78.79 (72.11)	F_2.43_ = 5.86 F = 0.27 F_2.43_ = 0.14	0.00 0.61 0.91
Mental Health TR _(*n* = 64)_ CR _(*n* = 55)_	66.88 (22.41) 72.00 (20.22)	76.63 (18.81) 79.93 (16.93)	79.69 (20.51) 79.27 (18.43)	78.38 (19.38) 81.38 (17.23)	F_2.57_ = 16.83 F = 0.91 F_2.57_ = 0.92	0.00 0.34 0.42
Vitality TR _(*n* = 64)_ CR _(*n* = 55)_	46.64 (28.27) 52.00 (25.58)	58.59 (25.55) 60.73 (23.64)	60.47 (26.20) 59.73 (25.83)	61.33 (26.71) 65.64 (23.27)	F_2.46_ = 18.47 F = 0.47 F_2.46_ = 0.90	0.00 0.50 0.43
Pain TR _(*n* = 64)_ CR _(*n* = 55)_	59.00 (27.47) 61.84 (28.63)	72.11 (28.79) 73.20 (26.86)	71.08 (29.04) 77.84 (26.35)	76.33 (27.31) 74.76 (25.21)	F_2.61_ = 13.90 F = 0.35 F_2.61_ = 0.86	0.00 0.56 0.45
General Health TR _(*n* = 64)_ CR _(*n* = 55)_	60.75 (23.81) 65.13 (21.28)	63.20 (25.27) 65.80 (20.50)	64.16 (25.24) 63.95 (21.13)	62.02 (25.24) 64.98 (22.56)	F_2.80_ = 0.35 F = 0.41 F_2.80_ = 0.71	0.77 0.53 0.54
